# Real-Time Biologically Inspired Action Recognition from Key Poses Using a Neuromorphic Architecture

**DOI:** 10.3389/fnbot.2017.00013

**Published:** 2017-03-22

**Authors:** Georg Layher, Tobias Brosch, Heiko Neumann

**Affiliations:** Institute of Neural Information Processing, Ulm UniversityUlm, Germany

**Keywords:** action recognition, key pose selection, deep learning, neuromorphic architecture, IBM neurosynaptic system

## Abstract

Intelligent agents, such as robots, have to serve a multitude of autonomous functions. Examples are, e.g., collision avoidance, navigation and route planning, active sensing of its environment, or the interaction and non-verbal communication with people in the extended reach space. Here, we focus on the recognition of the action of a human agent based on a biologically inspired visual architecture of analyzing articulated movements. The proposed processing architecture builds upon coarsely segregated streams of sensory processing along different pathways which separately process form and motion information (Layher et al., 2014). Action recognition is performed in an event-based scheme by identifying representations of characteristic pose configurations (key poses) in an image sequence. In line with perceptual studies, key poses are selected unsupervised utilizing a feature-driven criterion which combines extrema in the motion energy with the horizontal and the vertical extendedness of a body shape. Per class representations of key pose frames are learned using a deep convolutional neural network consisting of 15 convolutional layers. The network is trained using the *energy-efficient deep neuromorphic networks* (*Eedn*) framework (Esser et al., 2016), which realizes the mapping of the trained synaptic weights onto the *IBM Neurosynaptic System* platform (Merolla et al., 2014). After the mapping, the trained network achieves real-time capabilities for processing input streams and classify input images at about 1,000 frames per second while the computational stages only consume about 70 mW of energy (without spike transduction). Particularly regarding mobile robotic systems, a low energy profile might be crucial in a variety of application scenarios. Cross-validation results are reported for two different datasets and compared to state-of-the-art action recognition approaches. The results demonstrate, that (I) the presented approach is on par with other key pose based methods described in the literature, which select key pose frames by optimizing classification accuracy, (II) compared to the training on the full set of frames, representations trained on key pose frames result in a higher confidence in class assignments, and (III) key pose representations show promising generalization capabilities in a cross-dataset evaluation.

## 1. Introduction

Analyzing and understanding the actions of humans is one of the major challenges for future technical systems aiming at visual sensory behavior analysis. Acquiring knowledge about what a person is doing is of importance and sometimes even crucial in a variety of scenarios. In the context of automated surveillance systems, action analysis is an essential ability, allowing to identify potential threads emanating from an individual or a group of persons. In Human-Computer-Interaction (HCI), action analysis helps in understanding the objectives and intentions of a user and increases the potential of a system to adapt to the specific context of an interaction and appropriately support, guide or protect the user. Moreover, recognizing actions in the surrounding area is an integral part of interpreting the own situative context and environment, and thus is in particular crucial for mobile robotic systems which may find themselves embedded in a variety of different situations.

In the presented work, as the first main contribution, a *feature-driven* key pose selection method is proposed, which is driven by combining two features in the biological motion input, namely extrema in the temporal motion energy signal and the relative extent of a subject's pose. Such temporally defined features (from the motion stream) help to automatically select key pose representations. The use of these dynamic features has been motivated by psychophysical investigations (Thirkettle et al., 2009) which demonstrate that humans select specific poses in a continuous sequence of video input based on such criteria. We first show how such key poses define events within articulated motion sequences and how these can be reliably and automatically detected. The proposed processing architecture builds upon coarsely segregated streams of sensory processing along different pathways which separately process form and motion information (Giese and Poggio, 2003). An interaction between the two processing streams enables an automatic selection of characteristic poses during learning (Layher et al., 2014). To use such recognition functionality in an autonomous neurobiologically inspired recognition system various constraints need to be satisfied. Such neurobiological systems need to implement the underlying processes along the processing and recognition cascade which defines the parts of their cognitive functionality.

As the second key contribution, we employ here an *energy efficient deep convolutional neural network* (Eedn; Esser et al., 2016) to realize the key pose learning and classification, which achieves a computationally efficient solution using a sparse and energy efficient implementation based on neuromorphic hardware. This allows us to establish a cascaded hierarchy of representations with an increasing complexity for key pose form and motion patterns. After their establishment, key pose representations allow an assignment of a given input image to a specific action category. We use an offline training scheme that utilizes a deep convolutional neural network with 15 convolutional layers. The trained network runs on IBM's TrueNorth chip (Merolla et al., 2014; Akopyan et al., 2015). This solution renders it possible to approach faster than real-time capabilities for processing input streams and classify articulated still images at about 1, 000 frames per second while the computational stages consume only about 70 mW of energy. We present cross-validation results on an action recognition dataset consisting of 14 actions and 22 subjects and about 29, 000 key pose frames, which show a recall rate for the presented approach of about 88%, as well as a comparison to state-of-the-art action recognition approaches on a second dataset. To show the generalization capabilities of the proposed key pose based approach, we additionally present the results of a cross-dataset evaluation, where the training and the testing of the network was performed on two completely separate datasets with overlapping classes.

## 2. Related work

The proposed key pose based action recognition approach is motivated and inspired by recent evidences about the learning mechanisms and representations involved in the processing of articulated motion sequences, as well as hardware and software developments from various fields of visual sciences. For instance, empirical studies indicate, that special kinds of events within a motion sequence facilitate the recognition of an action. Additional evidences from psychophysics, as well as neurophysiology suggest that both, form and motion information contribute to the representation of an action. Modeling efforts propose functional mechanisms for the processing of biological motion and show how such processing principles can be transfered to technical domains. Deep convolutional networks make it possible to learn hierarchical object representations, which show an impressive recognition performance and enable the implementation of fast and energy efficient classification architectures, particularly in combination with neuromorphic hardware platforms. In the following sections, we will briefly introduce related work and results from different scientific fields, all contributing to a better understanding of action representation and the development of efficient action recognition approaches.

### 2.1. Articulated and biological motion

Starting with the pioneering work of Johansson (1973), perceptual sciences gained more and more insights about how biological motion might be represented in the human brain and what the characteristic properties of an articulated motion sequence are. In psychophysical experiments, humans show a remarkable performance in recognizing biological motions, even when the presented motion is reduced to a set of points moving coherently with body joints (*point light stimuli*; PLS). In a detection task, subjects were capable of recognizing a walking motion within about 200 ms (Johansson, 1976). These stimuli, however, are not free of – at least configurational – form information and the discussion about the contributions of form and motion in biological motion representation is still ongoing (Garcia and Grossman, 2008). Some studies indicate a stronger importance of motion cues (Mather and Murdoch, 1994), others emphasize the role of configurational form information (Lange and Lappe, 2006). Even less is known about the specific nature and characteristic of the visual cues which facilitate the recognition of a biological motion sequence. In Casile and Giese (2005), a statistical analysis as well as the results of psychophysical experiments indicate that local opponent motion in horizontal direction is one of the critical features for the recognition of PLS. Thurman and Grossman (2008) conclude, that there are specific moments in an action performance which are “more perceptually salient” compared to others. Their results emphasize the importance of dynamic cues in moments when the distance between opposing limbs is the lowest (corresponding to local opponent motion; maxima in the motion energy). On the contrary, more recent findings by Thirkettle et al. (2009) indicate, that moments of a large horizontal body extension (co-occurring with minima in the motion energy) facilitate the recognition of a biological motion in a PLS.

In neurophysiology, functional imaging studies (Grossman et al., 2000), as well as single-cell recordings (Oram and Perrett, 1994) indicate the existence of specialized mechanisms for the processing of biological motion in the *superior temporal sulcus* (STS). STS has been suggested to be a point of convergence of the separate dorsal “where” and the ventral “what” pathways (Boussaoud et al., 1990; Felleman and Van Essen, 1991), containing cells which integrate form and motion information of biological objects (Oram and Perrett, 1996) and selectively respond to, e.g., object manipulation, face, limb and whole body motion (Puce and Perrett, 2003). Besides the evidence that both form and motion information contribute to the registration of biological motion, action specific cells in STS are reported to respond to static images of articulated bodies which in parallel evoke activities in the *medio temporal* (MT) and *medial superior temporal* (MST) areas of the dorsal stream (*implied motion*), although there is no motion present in the input signal (Kourtzi and Kanwisher, 2000; Jellema and Perrett, 2003). In line with the psychophysical studies, these results indicate that poses with a specific feature characteristic (here, articulation) facilitate the recognition of a human motion sequence.

Complementary modeling efforts in the field of computational neuroscience suggest potential mechanisms which might explain the underlying neural processing and learning principles. In Giese and Poggio (2003) a model for the recognition of biological movements is proposed, which processes visual input along two separate form and motion pathways and temporally integrates the responses of prototypical motion and form patterns (snapshots) cells via asymmetric connections in both pathways. Layher et al. (2014) extended this model by incorporating an interaction between the two pathways, realizing the automatic and unsupervised learning of key poses by modulating the learning of the form prototypes using a motion energy based signal derived in the motion pathway. In addition, a feedback mechanism is proposed in this extended model architecture which (I) realizes sequence selectivity by temporal association learning and (II) gives a potential explanation for the activities in MT/MST observed for static images of articulated poses in neurophysiological studies.

### 2.2. Action recognition in image sequences

In computer vision, the term vision-based action recognition summarizes approaches to assign an action label to each frame or a collection of frames of an image sequence. Over the last decades, numerous vision-based action recognition approaches have been developed and different taxonomies have been proposed to classify them by different aspects of their processing principles. In Poppe (2010), action recognition methods are separated by the nature of the image representation they rely on, as well as the kind of the employed classification scheme. Image representations are divided into *global representations*, which use a holistic representation of the body in the *region of interest* (ROI; most often the bounding box around a body silhouette in the image space), and *local representations*, which describe image and motion characteristics in a spatial or spatio-temporal local neighborhood. Prominent examples for the use of whole body representations are *motion history images* (MHI) (Bobick and Davis, 2001), or the application of *histograms of oriented gradients* (HOG) (Dalal and Triggs, 2005; Thurau and Hlavác, 2008). Local representations are, e.g., employed in Dollar et al. (2005), where motion and form based descriptors are derived in the local neighborhood (cuboids) of spatio-temporal interest points. Classification approaches are separated into *direct classification*, which disregard temporal relationships (e.g., using histograms of prototype descriptors, Dollar et al., 2005) and *temporal state-space models*, which explicitly model temporal transitions between observations (e.g., by employing *Hidden Markov models* (HMMs) Yamato et al., 1992, or *dynamic time warping* (DTW) Chaaraoui et al., 2013). For further taxonomies and an exhaustive overview of computer vision action recognition approaches we refer to the excellent reviews in Gavrila (1999); Aggarwal and Ryoo (2011); Weinland et al. (2011).

The proposed approach uses motion and form based feature properties to extract key pose frames. The identified key pose frames are used to learn class specific key pose representations using a deep convolutional neural network (DCNN). Classification is either performed framewise or by temporal integration through majority voting. Thus, following the taxonomy of Poppe (2010), the approach can be classified as using *global representations* together with a *direct classification* scheme. Key pose frames are considered as temporal events within an action sequence. This kind of action representation and classification is inherently invariant against variations in (recording and execution) speed. We do not argue that modeling temporal relationships between such events is not necessary in general. The very simple temporal integration scheme was chosen to focus on an analysis of the importance of key poses in the context of action representation and recognition. Because of the relevance to the presented approach, we will briefly compare specifically key pose base action recognition approaches in the following.

### 2.3. Key pose based action recognition

Key pose based action recognition approaches differ in their understanding of the concept of key poses. Some take a phenomenological perspective and define key poses as events which possess a specific feature characteristic giving rise to their peculiarity. There is no a priori knowledge available about whether, when and how often such *feature-driven* events occur within an observed action sequence, neither during the establishment of the key pose representations during training, nor while trying to recognize an action sequence. Others regard key pose selection as the result of a statistical analysis, favoring poses which are easy to separate among different classes or maximally capture the characteristics of an action sequence. The majority of approaches rely on such statistical properties and either consider the *intra-* or the *inter-class* distribution of image-based pose descriptors to identify key poses in action sequences.

#### Intra-class based approaches

Approaches which evaluate *intra-class* properties of the feature distributions regard key poses as the most representative poses of an action and measures of centrality are exploited on agglomerations in pose feature spaces to identify the poses which are most common to an action sequence. In Chaaraoui et al. (2013), a contour based descriptor following (Dedeoğlu et al., 2006) is used. Key poses are selected by repetitive *k*-means clustering of the pose descriptors and evaluating the resulting clusters using a compactness metric. A sequence of nearest neighbor key poses is derived for each test sequence and dynamic time warping (DTW) is applied to account for different temporal scales. The class of the closest matching temporal sequence of key poses from the training set is used as the final recognition result. Based on histograms of oriented gradients (HOG) and histograms of weighted optical flow (HOWOF) descriptors, Cao et al. (2012) adapt a local linear embedding (LLE) strategy to establish a manifold model which reduces descriptor dimensionality, while preserving the local relationship between the descriptors. Key poses are identified by interpreting the data points (i.e., descriptors/poses) on the manifold as an adjacent graph and applying a *PageRank* (Brin and Page, 1998) based procedure to determine the vertices of the graph with the highest centrality, or relevance.

In all, key pose selection based on an *intra-class* analysis of the feature distribution has the advantage of capturing the characteristics of one action in isolation, independent of other classes in a dataset. Thus, key poses are not dataset specific and – in principle – can also be shared among different actions. However, most *intra-class* distribution based approaches build upon measures of centrality (i.e., as a part of cluster algorithms) and thus key poses are dominated by frequent poses of an action. Because they are part of transitions between others, frequent poses tend to occur in different classes and thus do not help in separating them. Infrequent poses, on the other hand, are not captured very well, but are intuitively more likely to be discriminative. The authors' are not aware of an *intra-class* distribution based method which tries to identify key poses based on their infrequency or abnormality (e.g., by evaluating cluster sizes and distances).

#### Inter-class based approaches

Approaches based on *inter-class* distribution, on the other hand, consider highly discriminative poses as key poses to separate different action appearances. Discriminability is here defined as resulting in either the best classification performance or in maximum dissimilarities between the extracted pose descriptors of different classes. To maximize the classification performance, Weinland and Boyer (2008) propose a method of identifying a vocabulary of highly discriminative pose exemplars. In each iteration of the forward selection of key poses, one exemplar at a time is added to the set of key poses by independently evaluating the classification performance of the currently selected set of poses in union with one of the remaining exemplars in the training set. The pose exemplar, which increases classification performance the most is then added to the final key pose set. The procedure is repeated until a predefined number of key poses is reached. Classification is performed based on a distance metric obtained by either silhouette-to-silhouette or silhouette-to-edge matching. Liu et al. (2013) combine the output of the early stages of an HMAX inspired processing architecture (Riesenhuber and Poggio, 1999) with a center-surround feature map obtained by subtracting several layers of a Gaussian pyramid and a wavelet laplacian pyramid feature map into framewise pose descriptors. The linearized feature descriptors are projected into a low-dimensional subspace derived by principal component analysis (PCA). Key poses are selected by employing an adaptive boosting technique (AdaBoost; Freund and Schapire, 1995) to select the most discriminative feature descriptors (i.e., poses). A test action sequence is matched to the thus reduced number of exemplars per action by applying an adapted local naive Bayes nearest neighbor classification scheme (LNBNN; McCann and Lowe, 2012). Each descriptor of a test sequence is assigned to its *k* nearest neighbors and a classwise voting is updated by the distance of a descriptor to the respective neighbor weighted by the relative number of classes per descriptor. In Baysal et al. (2010), noise reduced edges of an image are chained into a contour segmented network (CSN) by using orientation and closeness properties and transformed into a 2-adjacent segment descriptor (k-AS; Ferrari et al., 2008). The most characteristic descriptors are determined by identifying *k* candidate key poses per class using the *k*-medoids clustering algorithm and selecting the most distinctive ones among the set of all classes using a similarity measure on the 2-AS descriptors. Classification is performed by assigning each frame to the class of the key pose with the highest similarity and sequence-wide majority voting. Cheema et al. (2011) follow the same key pose extraction scheme, but instead of selecting only the most distinctive ones, key pose candidates are weighted by the number of false and correct assignments to an action class. A weighted voting scheme is then used to classify a given test sequence. Thus, although key poses with large weights have an increased influence on the final class assignment, all key poses take part in the classification process. Zhao and Elgammal (2008) use an information theoretic approach to select key frames within action sequences. They propose to describe the local neighborhood of spatiotemporal interest points using an intensity gradient based descriptor (Dollar et al., 2005). The extracted descriptors are then clustered, resulting in a codebook of prototypical descriptors (visual words). The pose prototypes are used to estimate the discriminatory power of a frame by calculating a measure based on the conditional entropy given the visual words detected in a frame. The frames with the highest discriminatory power are marked as key frames. Chi-square distances of histogram based spatiotemporal representations are used to compare key frames from the test and training datasets and majority voting is used to assign an action class to a test sequence.

For a given pose descriptor and/or classification architecture, *inter-class* based key pose selection methods in principle minimize the recognition error, either for the recognition of the key poses (e.g., Baysal et al., 2010; Liu et al., 2013) or for the action classification (e.g., Weinland and Boyer, 2008). But, on the other hand, key poses obtained by *inter-class* analysis inherently do not cover the most characteristic poses of an action, but the ones which are the most distinctive within a specific set of actions. Applying this class of algorithms on two different sets of actions sharing one common action might result in a different selection of key poses for the same action. Thus, once extracted, key pose representations do not necessarily generalize over different datasets/domains and, in addition, sharing of key poses between different classes is not intended.

#### Feature-driven approaches

*Feature-driven* key pose selection methods do not rely on the distribution of features or descriptors at all and define a key pose as a pose which co-occurs with a specific characteristic of an image or feature. Commonly employed features, such as extrema in a motion energy based signal, are often correlated with pose properties such as the degree of articulation or the extendedness. Compared to statistical methods, this is a more pose centered perspective, since parameters of the pose itself are used to select a key pose instead of parameters describing the relationship or differences between poses.

Lv and Nevatia (2007) select key poses in sequences of 3D-joint positions by automatically locating extrema of the motion energy within temporal windows. Motion energy in their approach is determined by calculating the sum over the L^2^ norm of the motion vectors of the joints between two temporally adjacent timesteps. 3D motion capturing data is used to render 2D projections of the key poses from different view angles. Single frames of an action sequence are matched to the silhouettes of the resulting 2D key pose representations using an extension of the Pyramid Match Kernel algorithm (PMK; Grauman and Darrell, 2005). Transitions between key poses are modeled using action graph models. Given an action sequence, the most likely action model is determined using the Viterbi Algorithm. In Gong et al. (2010), a key pose selection mechanism for 3D human action representations is proposed. Per action sequence, feature vectors (three angles for twelve joints) are projected onto the subspace spanned by the first three eigenvectors obtained by PCA. Several instances of an action are synchronized to derive the mean performance (in terms of execution) of an action. Motion energy is then defined by calculating the Euclidean distance between two adjacent poses in the mean performance. The local extrema of the motion energy are used to select the key poses, which after their reconstruction in the original space are used as the vocabulary in a bag of words approach. During recognition, each pose within a sequence is assigned to the key pose with the minimum Euclidean distance resulting in a histogram of key pose occurrences per sequence. These histograms serve as input to a support vector machine (SVM) classifier. In Ogale et al. (2007), candidate key poses are extracted by localizing the extrema of the mean motion magnitude in the estimated optical flow. Redundant poses are sorted out pairwise by considering the ratio between the intersection and the union of two registered silhouettes. The final set of unique key poses is used to construct a probabilistic context-free grammar (PCFG). This method uses an *inter-class* metric to reject preselected key pose candidates and thus is not purely feature-driven.

*Feature-driven* key pose selection methods are independent of the number of different actions within a dataset. Thus, retraining is not necessary if, e.g., a new action is added to a dataset and the sharing of key poses among different actions is in principle possible. Naturally, there is no guarantee, that the selected poses maximize the separability of pose or action classes.

## 3. Model/methods

To realize an energy efficient implementation for key pose based action recognition, the proposed model uses a neuromorphic deep convolutional neural network (DCNN) to selectively learn representations of key poses which are assigned to different action classes. In the preprocessing phase, optical flow is calculated on the input sequences and key pose frames are selected in an unsupervised manner. Form and motion information is calculated for each key pose frame. The concatenated form and motion information is then used as the input to the DCNN. In the following, detailed information about the image preprocessing, the key pose selection automatism and the structure and functionality of the DCNN are presented. All simulations were carried out using a neuromorphic computing paradigm and mapped to the IBM TrueNorth hardware platform (Merolla et al., 2014).

### 3.1. Key pose selection and image preprocessing

During preprocessing, two elementary processing steps are performed. First, the key pose selection is performed by automatically analyzing simple motion and form parameters. Second, the final input to the network is calculated by combining the form and motion representations *I*^form^ and *I*^motion^ obtained by simple image-based operations.

#### Key pose selection

The key pose selection process operates upon two parameters, namely (I) local temporal extrema in the motion energy and (II) the extendedness of a subject at a given timestep. Optical flow is calculated using a differential method, as suggested in the *Lucas-Kanade* optical flow estimation algorithm (Lucas and Kanade, 1981). Given an image sequence *I*(**x**, *t*), the optical flow **u**(**x**, *t*) = (*u*(**x**, *t*), *v*(**x**, *t*)) at timestep *t* and position **x** = (*x, y*) is estimated in a local neighborhood *N*(**x**) by minimizing

(1) $$\sum_{y\in N(x)}W{\left(x-y\right)}^{2}{\left[I_{x}(y,t)u(x,t)+I_{y}(x,t)v(x,t)+I_{t}(y,t)\right]}^{2},$$

where *W*(**x** − **y**) increases the influence of the optical flow constraints within the center of the local neighborhood (for details see Barron et al., 1994). The spatiotemporal derivatives *I*_*x*_, *I*_*y*_ and *I*_*t*_ are estimated by convolution of the image sequences with the forth-order central difference [−1, 8, 0, −8, 1]/12 and it's transpose in the spatial and the first-order backward difference [−1, 1] in the temporal domain. A separable 2D kernel with 1D coefficients of [1, 4, 6, 4, 1]/16 is used to realize the weighted integration of the derivatives within a 5 × 5 spatial neighborhood (*N*(**x**))^1^. The use of the *Lucas-Kanade* algorithm is not a hard prerequisite for the proposed approach. Other types of optical flow estimators might be applied as well (e.g., (Brosch and Neumann, 2016), which is capable to be executed on neuromorphic hardware). The overall motion energy *E*^flo^ is then calculated by integrating the speed of all estimated flow vectors within the vector field.

(2) $$E^{\text{flo}}(t)=\sum_{x\in I(x,t)}\|u(x,t){\|}_{2}=\sum_{x\in I(x,t)}\sqrt{u{\left(x,t\right)}^{2}+v{\left(x,t\right)}^{2}},$$

Motion energy is smoothed by convolving the estimated motion energy with a Gaussian kernel, ${Ẽ}^{\text{flo}}\left(t\right)=\left(E^{\text{flo}}*G_{\sigma }\right)\left(t\right)$. In the performed simulations, σ = 2 and σ = 4 were used dependent on the dataset^2^. Potential key pose frames are then marked by identifying the local extrema of the motion energy signal.

(3) $$K^{\text{flo}}=\{I(t),t\in [1,\ldots ,T]|\text{t is a local extremum of }{\tilde{E}}^{\text{flo}}(t)\},$$

The relative horizontal and vertical extent of a given pose at time *t* is then used to reject local extrema with an extent smaller than a predefined percentual threshold λ, as defined by:

(4) $$K=K^{\text{flo}}\cap K^{\text{ext}}.$$

with

(5) $$\begin{array}{l} K^{\text{ext}}=\{I(t),t\in [1,\ldots ,T]|({\text{Ext}}^{\text{ver}}(t)>(1+\lambda ){\bar{\text{Ext}}}^{\text{ver}})\\ \;\vee \;({\text{Ext}}^{\text{ver}}(t)<(1-\lambda ){\bar{\text{Ext}}}^{\text{ver}})\\ \;\vee \;({\text{Ext}}^{\text{hor}}(t)>(1+\lambda ){\bar{\text{Ext}}}^{\text{hor}})\\ \;\vee \;({\text{Ext}}^{\text{hor}}(t)<(1-\lambda ){\bar{\text{Ext}}}^{\text{hor}})\} \end{array}$$

**Figure 1:**
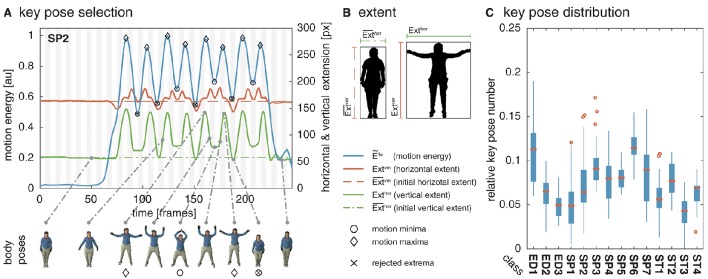
**Key pose selection**. For each action sequence, key pose frames are selected by identifying minima and maxima in the motion energy which co-occur with a sufficiently increased extent of the body pose. In **(A)**, the smoothed motion energy Ẽ^flo^ (blue) together with the horizontal (red) and vertical (green) extent of the body pose Ext^hor^ and Ext^ver^ and their reference values ${\bar{\text{Ext}}}^{\text{ver}}$ and ${\bar{\text{Ext}}}^{\text{hor}}$ (dashed, dash-dotted) are displayed for one of the actions used as part of the simulations (*SP2*/jumping jack). At the bottom, body poses for several frames are shown. Local minima in the motion energy are marked by a circle, local maxima by a diamond. Extrema which are rejected as a key pose frame because of an insufficient extent are additionally marked with ×. **(B)** Shows an example for the horizontal and vertical extent of a neutral and a highly articulated body pose. The first frame of each action sequence is defined to be the neutral pose. **(C)** Shows the relative number of identified key poses per action sequence for the uulmMAD dataset used for the simulations (see Section 4.1). Written informed consent for the publication of the exemplary images was obtained from the displayed subject.

In the performed simulations, values of λ = 0.1 and λ = 0.05 were used for the two different datasets. The percentual thresholds were determined manually with the aim to compensate for differences in the temporal resolution of the datasets. The horizontal and vertical extent Ext^hor^ and Ext^ver^ are derived framewise by estimating the width and the height of the bounding box enclosing the body shape. The extent of a neutral pose is used as the reference extent ${\bar{\text{Ext}}}^{\text{hor}}$ and ${\bar{\text{Ext}}}^{\text{ver}}$, which are derived from the width and height of the bounding box in the first frame of a sequence. Silhouette representations, and thus the bounding boxes of the bodies, are available for both datasets used in the simulations. In constrained recording scenarios, silhouettes can be extracted by background subtraction or using the optical flow fields calculated for the selection of the key pose frames. Figure 1A shows the motion energy signal Ẽ^flo^ together with the extent Ext^hor^ and Ext^ver^ and their reference values. A strong correlation between the motion energy and the extent of the pose can be seen. In Figure 1B, examples for the horizontal and the vertical extent are displayed for a neutral and a extended posture. While the motion energy allows an identification of temporal anchor points in a motion sequence, the extent helps in selecting the most characteristic ones.

#### Form and motion representations

**Figure 2:**
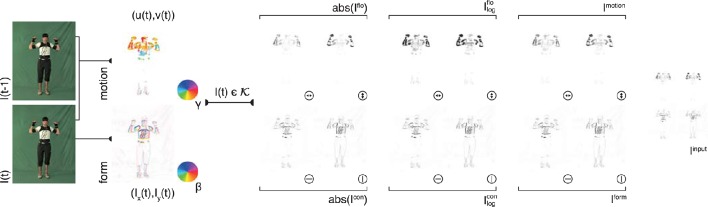
**Input generation**. Each frame of an action sequence is transformed into a combined motion and form representation. In the top row, the estimated optical flow is displayed in the second column (direction γ color-encoded) for two consecutive frames (first column). The optical flow field is then separated into horizontal and vertical components (third column) and their absolute value is log transformed (forth column) and normalized (fifth column). Form representations (bottom row) are derived framewise by estimating the horizontal and vertical derivatives *I*_*x*_ and *I*_*y*_ (second column, gradient orientation with polarity β color-encoded). The resulting contrast images are then log-transformed and normalized. The form and motion representations are combined into a single feature map *I*^input^ which is then fed into the convolutional neural network. Image sizes are increased for a better visibility. Written informed consent for the publication of exemplary images was obtained from the shown subject.

For each selected key pose frame $I^{\text{key}}\in K$, a form representation is derived by estimating the spatial derivatives $I_{x}^{\text{key}}$ and $I_{y}^{\text{key}}$ and combining them into one contour representation *I*^con^ by concatenating the orientation selective maps (see Figure 2, second row). The final form representation is then obtained by applying a logarithmic transformation emphasizing low range values and normalizing the response amplitudes, using the transformation:

(6) $$I_{\text{log}}^{\text{con}}=\log(1+5|I^{\text{con}}|)$$

(7) $$I^{\text{form}}=\frac{I_{\text{log}}^{\text{con}}}{\max(I_{\text{log}}^{\text{con}})}$$

Likewise, for each key pose frame *I*^key^, optical flow is separated into vertical and horizontal components and concatenated (see Figure 2, first row). The resulting motion representation *I*^flo^ is log-transformed and normalized. As for the contrast mapping, the transformation is given through:

(8) $$I_{\text{log}}^{\text{flo}}=\log(1+5|I^{\text{flo}}|))$$

(9) $$I^{\text{motion}}=\frac{I_{\text{log}}^{\text{flo}}}{\max(I_{\text{log}}^{\text{flo}})}$$

The form representations *I*^form^ and the motion representations *I*^motion^ are combined to an overall input representation *I*^input^ (Figure 2, last column). *I*^input^ is then used as an input for the training of the DCNN described in the following section.

### 3.2. Learning of class specific key pose representations

A neuromorphic deep convolutional neural network was used to establish classwise representations of the preselected and suppress wrapping key pose frames using a supervised learning scheme. The network was implemented using the *energy-efficient deep neuromorphic networks* (*Eedn*) framework (Esser et al., 2016), which adapts and extends the training and network functions of the *MatConvNet* toolbox (Vedaldi and Lenc, 2015). In the following for readers' convenience, we will briefly recapitulate and summarize key aspects of the framework and its extensions presented in Esser et al. (2016). In the framework, the weights established through learning match the representation scheme and processing principles used in neuromorphic computing paradigms. The structure of the DCNN follows one of the network parameter sets presented by Esser et al. (2016), which show a close to state-of-the-art classification performance on a variety of image datasets and allow the trained network to be run on a single IBM TrueNorth chip (Merolla et al., 2014).

A deep convolutional neural network is typically organized in a feedforward cascade of layers composed of artificial neurons (LeCun et al., 2010), which process the output of the proceeding layer (afferent synaptic connections) and propagate the result to the subsequent one (efferent synaptic connections). Following the definition in Esser et al. (2016), an artificial cell *j* in a DCNN calculates a weighted sum over the input to that cell, as defined by:

(10) $$s_{j}=\sum_{xy}\sum_{f}in_{xyf}w_{xyfj},$$

where *in*_*xyf*_ are the signals in the input field of cell *j* at locations (*xy*) in the spatial and (*f*) in the feature domain and, *w*_*xyfj*_ the respective synaptic weights. In the following, we will use the linear index *i* to denote locations in the (*xyf*) space-feature cube. Normalizing the weighted sum over a set of input samples (batch normalization) allows to accelerate the training of the network by standardizing *s*_*j*_ as defined through:

(11) $${\tilde{s}}_{j}=\frac{s_{j}-\mu_{j}}{\sigma_{j}+\epsilon }+b_{j},$$

with ${\tilde{s}}_{j}$ the standardized weighted sum, μ_*j*_ the mean and σ_*j*_ the standard deviation of *s* calculated over the number of training examples within a batch (Ioffe and Szegedy, 2015). *b*_*j*_ is a bias term, allowing to shift the activation function ϕ(•), and ϵ guarantees numerical stability. The output activation of the artificial neuron is calculated by applying an activation function on the standardized filter response:

(12) $$r_{j}=\phi ({\tilde{s}}_{j}).$$

Weight adaptation is performed through gradient descent by applying error backpropagation with momentum (Rumelhart et al., 1986). In the forward phase, an input pattern is propagated through the network until the activations of the cells in the output layer are obtained. In the backward phase, the target values of an input pattern are used to calculate the cross entropy *C* given the current and the desired response of the output layer cell activations, as defined by:

(13) $$C=-\sum_{j=1}^{M}v_{j}\ln(r_{j})=-\sum_{j=1}^{M}v_{j}\ln(\phi ({\tilde{s}}_{j})),$$

with *M* denoting the number of cells in the output layer. Here, *v*_*j*_ is the one-hot encoded target value (or teaching signal) of a cell *j* with activation *r*_*j*_. A *softmax* function is employed as activation function in the output layer, as defined through:

(14) $$\phi ({\tilde{s}}_{j})=\frac{e^{{\tilde{s}}_{j}}}{\sum_{k=1}^{M}e^{{\tilde{s}}_{k}}}.$$

The cross entropy error *E*(*t*) = *C* is then propagated backwards through the network and the synaptic weight adaptation is calculated for all cells in the output and hidden layers by applying the chain rule. The strength of weight adaptation Δ*w*_*ij*_ is given through:

(15) $$\Delta w_{ij}(t)=-\eta \frac{\partial E(t)}{\partial w_{ij}}+\alpha \Delta w_{ij}(t-1)=-\eta \delta_{j}in_{i}+\alpha \Delta w_{ij}(t-1),$$

(16) $$\text{with }\delta_{j}=\{\begin{array}{ll} \left(r_{j}-v_{j}\right) & \text{if }j\text{ is a neuron in the output layer}\\ \phi^{\prime }({\tilde{s}}_{j})\sum_{k}\delta_{k}w_{jk} & \text{if }j\text{ is a neuron in a hidden layer}, \end{array}$$

which includes a momentum term for smoothing instantaneous weight changes. Here, *k* is the index of cells in the layer succeeding cell *j*, *t* describes the current training step, or iteration, and η denotes the learning rate. The momentum factor 0 ≤ α ≤ 1 helps the network to handle local minima and flat plateaus on the error surface. After the backward pass, weights are finally adapted by:

(17) $$w_{ij}(t+1)=w_{ij}(t)+\Delta w_{ij}(t).$$

To ensure the compatibility to neuromorphic processing principles, a binary activation function $\phi \left({\tilde{s}}_{j}\right)$ is applied in the hidden layers (for details see Section 3.3).

Within a convolutional layer, weights *w*_*ij*_ of a cell *j* are shared over multiple input fields, which are arranged as a regular grid in the source layer. The calculation of the weighted sum during the forward, as well as the integration of the error derivative during the backward pass can be formulated as a convolution with the input from the source, or the error signal from the succeeding layer. The weights *w*_*ij*_ act as the filter (or convolution) kernel, ${\tilde{s}}_{j}$ as the filter response and *r*_*j*_ as the output of an artificial cell. The *size* and *stride* of a filter allow to adjust the size and the overlap of the input fields to a filter in the source layer. A small stride results in an increased overlap and thus a large number of output values. The number of *features* defines how many different filters are employed in a layer. The weight matrices of the cells within a layer can be separated into *groups* of filters, which define the set of input features from the source layer covered by a filter^3^.

It is a common practice to construct deep neural networks by employing convolutional layers for feature extraction in the lower layers and connect them with (one or more) fully connected layers (equivalent to *Multilayer Perceptrons*/MLPs) on top for classification purposes. In contrast, the proposed network follows the strategy of *global average pooling* (gap) proposed in Lin et al. (2013) and applied in Esser et al. (2016). In the final convolutional layer of the network, one feature map is generated for each category of the classification problem. Instead of a full connectivity, the average value of each class-associated feature map is propagated to the output (softmax) layer. Due to their association to classes, the feature maps can directly be interpreted as confidence maps. Following the softmax layer, the cross-entropy error is calculated using one-hot encoded target values *v*_*j*_ and propagated back through the network (according to Equation 16). Networks using parameter-free *global average pooling* layers in combination with softmax are less prone to overfitting (compared to MLPs) and increase the robustness to spatial translations (for details see Lin et al., 2013).

**Figure 3:**
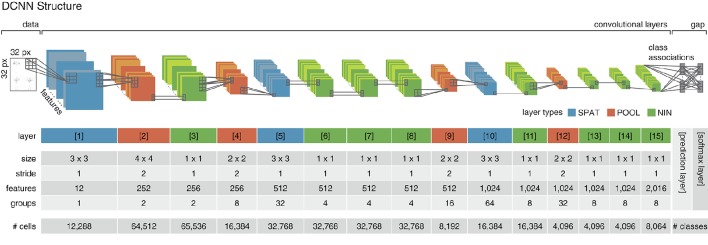
**Deep convolutional neural network structure**. The implemented DCNN follows the structure proposed in Esser et al. (2016) and employs three different convolutional layer types (layers 1–15). *Spatial layers* (SPAT; colored in blue) perform a linear filtering operation by convolution. *Pooling layers* (POOL; colored in red) decrease the spatial dimensionality while increasing the invariance and diminishing the chance of overfitting. *Network-in-network* layers (NIN; colored in green) perform a parametric cross channel integration (Lin et al., 2013). The proposed network consists of a data (or input) layer, 15 convolutional layers and a prediction and softmax layer on top. Each of the cells in the last convolutional layer (layer 15) is associated with one class of the classification problem. In the prediction layer, the average class-associated activations are derived (*global average pooling*/gap) and fed into the softmax layer (i.e., one per class), where the cross-entropy error is calculated and propagated backwards through the network. The parameters used for the convolutional layers of the network are given in the central rows of the table. In the last row, the number of artificial cells per layer is listed. The cell count in the *prediction* and *softmax* layer depends on the number of categories of the classification task (i.e., the number of actions in the dataset).

The employed network consists of 15 convolutional layers, which implement three different types of convolutional operations. *Spatial* layers (SPAT) perform a standard convolution operation, *pooling* layers (POOL) reduce the spatial dimensions by applying a convolution with a large stride (Springenberg et al., 2014), *network-in-network* layers (NIN) are realized by convolutional layers with a size of 1*x*1 and a stride of 1 and act as cross channel integration layers (Lin et al., 2013). The network structure is summarized in Figure 3. Each of the cells in the last convolutional layer (layer 15) is assigned to one class. During learning, activities in this layer are averaged per feature map and fed into the *softmax* layer. For recognition, the average output of the cell populations associated to the individual classes are used as prediction values and serve as the final output $r_{c}^{\text{class}}$ of the network (*prediction* layer in Figure 3).

### 3.3. Neuromorphic implementation

Processing actual spikes in hardware, the execution of a DCNN on a neuromorphic platform poses several constraints on the activity and weight representation schemes. Since the processing architecture of the TrueNorth neuromorphic platform is based on event-based representations, the gradual activations need to be mapped onto a spike-based mechanism. To be in conformity with these processing principles, Esser et al. (2016) employ a binary activation function, as defined by:

(18) $$\phi ({\tilde{s}}_{j})=\{\begin{array}{ll} 1 & \text{if }\tilde{s_{j}}\geq 0\\ 0 & \text{otherwise}, \end{array}$$

and ternary synaptic weights (*w*_*xyf*_ ∈ {−1, 0, 1}). For the backpropagation of the error signal, the derivative of the binary activation is approximated linearly in the range of [0, 1], as given through:

(19) $$\frac{\partial \phi ({\tilde{s}}_{j})}{\partial {\tilde{s}}_{j}}\approx \text{max}(0,1-\left|{\tilde{s}}_{j}\right|).$$

During training, a copy of the model weights is held in a *shadow network*, which allows gradual weight adaptation. Weight updates are performed on values in the shadow network using high precision values. For the forward and backward pass, the hidden weights $w_{ij}^{h}$ in the shadow network are clipped to [−1, 1] and mapped to the ternary values using rounding and hysteresis, following:

(20) $$w_{ij}(t)=\{\begin{array}{ll} -1 & \text{if}w_{ij}^{h}(t)\leq -0.5-h\\ 0 & \text{if}w_{ij}^{h}(t)\geq -0.5+h\wedge w_{ij}^{h}(t)\leq 0.5-h\\ 1 & \text{if}w_{ij}^{h}(t)\geq 0.5+h\\ w_{ij}(t-1) & \text{otherwise} \end{array}$$

(for details refer to Esser et al., 2016). The hidden weights $w_{ij}^{h}$ allow the synaptic connection strengths to switch between the ternary values based on small changes in the error gradients obtained during backpropagation, while the hysteresis factor *h* prevents them from oscillating. The parameters for the training of the network were chosen according to Esser et al. (2016), using a momentum factor of α = 0.9 and a learning rate of η = 20 (reduced by a factor of 0.1 after 2/3 and 5/6 of the total training iterations). The hysteresis factor *h* was set to 0.1. The mapping of the training network on the TrueNorth platform was performed by the *Eedn* framework. Training was carried out on Nvidia GPUs, testing was performed on the IBM TrueNorth NS1e board.

The IBM TrueNorth chip consists of 4, 096 interconnected neurosynaptic cores with 1 million spiking neurons and 256 million configurable synaptic connections. For the execution of the network on the TrueNorth chip, the trained network parameters are mapped to hardware using an abstraction of a TrueNorth program called *Corelet* (Amir et al., 2013). The platform independent *Corelets* translate the network parameters into a TrueNorth specific configuration, which can be used to program the parameters of the neurons and synaptic connection strengths on the chip. For details on *Corelets* and the mapping of the DCNN on neuromorphic hardware platforms refer to Amir et al. (2013); Esser et al. (2016).

### 3.4. Temporal integration of framewise class predictions

After the training of the DCNN, classification is either performed framewise by directly selecting the class corresponding to the cell population in layer 15 with the maximum average activation, or by integrating the individual framewise classification results using majority voting in temporal windows or over the full sequence.

For framewise classification, a key pose frame is identified in an input image sequence *I*(**x**, *t*) and preprocessed as described in Section 3.1. The resulting input map *I*^input^ is fed into the DCNN and the class label *c* associated to the cell population in layer 15 with the maximum average output $r_{c}^{\text{class}}$ defines the class prediction for *I*^input^. The value of $r_{c}^{\text{class}}$ can directly be interpreted as the confidence in the prediction.

In sliding window based classification, the predicted class labels for key pose frames are collected within temporal windows of size *n*[frames], which are shifted over the input sequence *I*(**x**, *t*). The class with the most frequent occurrence of key pose frames determines the class predicted for the window (majority voting). At the moment, we do not use the confidence $r_{c}^{\text{class}}$ of the predictions as weights for the voting. Note that it is not guaranteed, that key pose frames occur in all temporal windows. Windows which do not contain key poses are not used for evaluation.

Full sequence classification follows the same principle as sliding window based classification, but collects all key pose frames within a sequence. Thus, the amount of temporal information integrated in the voting process might differ substantially from sequence to sequence.

## 4. Datasets

The proposed action recognition approach was evaluated using two different action datasets. Due to the higher number of subjects and actions, we focused our analysis on the *uulm multiperspective action dataset* (uulmMAD). In addition, we analyzed the performance on the widely used *Weizmann* dataset to allow a comparison to other approaches and to perform a cross-dataset evaluation of overlapping classes. In the following, we will briefly describe the main characteristics of the two datasets.

### 4.1. uulmMAD

**Figure 4:**
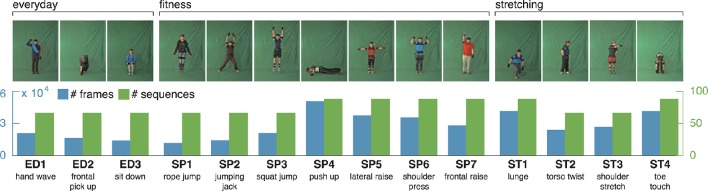
**uulmMAD – uulm multiperspective action dataset**. The uulmMAD dataset contains 14 actions in the area of everyday activities, fitness/sports and stretching performed by 31 subjects. Per subject, eight of the actions are repeated three times, six actions are performed four times with varying speed. Actions were recorded by three synchronized cameras (frontal, diagonal and lateral) with a frame rate of 30 Hz and an inertial motion capturing system with a sample rate of 120 Hz. Silhouettes were extracted using chromakeying. At the time we carried out the simulations, silhouettes were available for 22 subjects. In the first row exemplary pictures are shown for all actions. The number of videos (green) and total sum of frames (blue) which were available for the evaluation are displayed in the second row. At the bottom, an abbreviation for each action is defined and a short description is given. Written informed consent for the publication of exemplary images was obtained from the displayed subjects.

The *uulm multiperspective action dataset*^4^ (uumlMAD; Glodek et al., 2014) consists of data from 31 subjects performing actions from the areas of everyday life (ED), sport/fitness (SP) and stretching (ST). Eight of the actions are repeated three times, six actions are performed four times with varying speed. Altogether, each action is performed either 93 or 124 times. Actions were recorded in front of a greenscreen using three synchronized cameras and the body posture was captured in parallel by an inertial motion capturing system worn by the subjects. To decrease the likelihood of similar visual appearances, the motion capture suit was covered by additional clothes whenever possible. Figure 4 shows the 14 actions together with a characteristic picture, an abbreviation and a short description for each action.

At the time we carried out the simulations, silhouette representations were available for all sequences of 22 subjects. Since the silhouettes are used to calculate an estimate of the horizontal and vertical extent of a pose, only the frontal recordings of this subset of subjects were used within the evaluation. Some action pairs (e.g., ED2 and ST4) in the dataset are deliberately intended to appear visually similar and thus be difficult to separate. In total, the sequences used for evaluation contain 381, 194 frames, of which 28, 902 are selected by the key pose selection procedure.

### 4.2. Weizmann dataset

**Figure 5:**
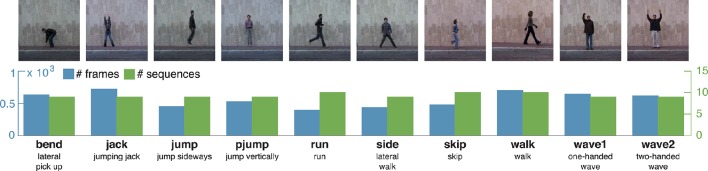
**Weizmann action dataset**. The Weizmann dataset (Gorelick et al., 2007) is one of the most commonly used action recognition datasets and consist of ten actions, recorded for nine subjects. Actions are performed once (occasionally twice) per subject in front of a static background. Silhouette representations are provided for all sequences. Representative images are displayed alongside with the number of frames and sequences, a label and a short description per class.

To allow a comparison with different action recognition approaches, simulations were additionally carried out using a well established action dataset. The *Weizmann dataset*^5^ (see Figure 5; Gorelick et al., 2007) consists of ten actions performed by nine subjects. Actions are mostly performed once per subject, although some actions are occasionally performed twice. Actions are captured in 25*Hz* from a frontoparallel perspective in front of a uniform background.

Silhouettes are available for all subjects and sequences. In total, the sequences contain 5, 594 frames, 1, 873 of which are identified as key pose frames by using the procedure described in Section 3.1.

## 5. Results

Several simulations were carried out to evaluate the performance of the proposed key pose based action recognition approach. The simulations were intended to address questions related to (I) the overall performance of the approach on different datasets using a framewise, as well as windowed and full sequence majority voting recognition schemes, (II) a comparison to other action recognition methods, (III) a juxtaposition of key pose based and full sequence learning, and (IV) cross-dataset evaluation. Since action recognition datasets—in particular, in case of framewise recognition—are often highly imbalanced, we provide different types of performance measures, as well as classwise performance values for the most essential results. Since the nomenclature and definition of performance measures vary largely in the pattern recognition and machine learning community we will briefly define and describe the reported measures to allow a better comparability. For a comprehensive discussion on performance measures, we refer to Sokolova and Lapalme (2009) and the contributions of D. Powers, e.g. (Powers, 2013).

In a multiclass classification problem with *N* classes tp_*i*_ (true positives) are commonly defined as the number of correct acceptances (hits) for a class C_*i*_ (*i* ∈ [1, …, *N*]), fn_*i*_ as the number of false rejections (misses), tn_*i*_ as the number of correct rejections of samples of different classes C_*j*≠*i*_ and fn_*i*_ (false negatives) as the number of false acceptances (false alarms). Together, these four counts constitute the confusion matrix and allow to derive a variety of measures describing the performance of a trained classification system. The ones used for the evaluation of the presented results are listed alongside with an abbreviation and their definition in Table 1.

**Table 1:** **Performance measures**.

**Measure**	**Abbreviation**	**Definition**
Recall	Rec_M_	$\frac{1}{N}\overset{N}{\underset{i=1}{\sum }}\frac{{\text{tp}}_{i}}{{\text{tp}}_{i}+{\text{fn}}_{i}}$
Informedness	Inf_M_	$\overset{N}{\underset{i=1}{\sum }}\frac{{\text{tp}}_{i}+{\text{fp}}_{i}}{{\text{tp}}_{i}+{\text{fn}}_{i}+{\text{tn}}_{i}+{\text{fp}}_{i}}\times \left(\frac{{\text{tp}}_{i}}{{\text{tp}}_{i}+{\text{fn}}_{i}}+\frac{{\text{tn}}_{i}}{{\text{fp}}_{i}+{\text{tn}}_{i}}-1\right)$
Markedness	Mark_M_	$\overset{N}{\underset{i=1}{\sum }}\frac{{\text{tp}}_{i}+{\text{fn}}_{i}}{{\text{tp}}_{i}+{\text{fn}}_{i}+{\text{tn}}_{i}+{\text{fp}}_{i}}\times \left(\frac{{\text{tp}}_{i}}{{\text{tp}}_{i}+{\text{fp}}_{i}}+\frac{{\text{tn}}_{i}}{{\text{fn}}_{i}+{\text{tn}}_{i}}-1\right)$
Matthews Correlation	MCC_M_	$\pm \sqrt{{\text{Mark}}_{\text{M}}\times {\text{Inf}}_{\text{M}}}$

All multiclass performance measures are calculated using macro averaging (M), since using micro averaging, classes with a large number of examples would dominate the averaging. Rec_M_, often referred to as (average) recognition rate or somewhat misleading as (classification) accuracy, might be the performance measurement most frequently used in the action recognition literature and describes the average percentage of correctly identified positive examples per class. Inf_M_ reflects how informed the decision of a classifier is in comparison to chance, whereas Mark_M_ follows the inverse concept by describing how likely the prediction variable is marked by the true variable (Powers, 2013). Note, that when calculating the average per class values of Inf_M_ and Mark_M_ are weighted by the ${\text{Bias}}_{i}=\frac{{\text{tp}}_{i}+{\text{fp}}_{i}}{{\text{tp}}_{i}+{\text{fn}}_{i}+{\text{tn}}_{i}+{\text{fp}}_{i}}$ and the ${\text{Prevalence}}_{i}=\frac{{\text{tp}}_{i}+{\text{fn}}_{i}}{{\text{tp}}_{i}+{\text{fn}}_{i}+{\text{tn}}_{i}+{\text{fp}}_{i}}$, respectively. The Matthews Correlation Coefficient MCC_M_ can be derived by calculating the geometric mean of Inf_M_ and Mark_M_ and expresses the correlation between predicted classes and true values.

Leave-one-subject-out cross-validation (LOSO) was performed in all test scenarios and the resulting average performance measures are reported together with the corresponding standard deviations. In the following, rates are either reported in a range of [0, 100] or [0, 1] (due to limited space).

### 5.1. Classification performance

**Figure 6:**
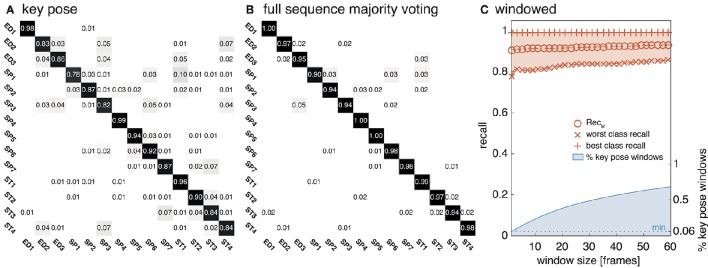
**uulmMAD classification performance**. The proposed network was trained on the key pose frames extracted from the uulmMAD action recognition dataset. **(A)** Shows the per class classification rates obtained by single key pose frame classification. This allows the recognition of an action in the instant a key pose frame emerges in the input sequence. Average classwise recall (on the diagonal) ranges from 0.78 to 0.98. Some of the notable confusions between classes can be explained by a large visual similarity (e.g., between ED2 and ST4). In **(B)** sequence level majority voting was applied. The final decision is made after evaluating all key pose frames within an action sequence and determining the class with the most frequent occurrence of key poses. The resulting per class values of Rec_M_ range from 0.94 to 1.00. A sliding window based classification scheme was evaluated in **(C)**. The best and worst per class average recall values together with the average value of Rec_M_ are displayed for temporal window sizes from 1 to 60 frames. In addition, the percentage of windows containing one or more key pose frames (and thus allow a classification of the action) is shown (blue line).

The equivalent network structure (see Section 3.2) was used to train the network on the two datasets described in Section 4. In case of the uulmMAD dataset, 28, 902 key pose frames (per class average 2, 064.43, std 1, 097.16) were selected and used as the training input. 576 cells in the last convolutional layer (layer 15) of the CNN were assigned to each of the 14 classes in the dataset. The network was trained in 150, 000 iterations. Testing was performed using the preselected key pose frames of the test subject as input. The average population activation of the cells assigned to each class was used to infer the final classification decision (for an exemplary activation pattern see Figure **8**). Figure 6 summarizes classification results obtained for different temporal integration schemes of single frame classification results. A framewise classification scheme allows to recognize an action in an instant when the key pose frame is presented to the network. This kind of immediate decision might be crucial for systems which rely on decisions in real time. Not only the processing speed, but also the time necessary to sample and construct the action descriptors is relevant in this context. Figure 6A summarizes the framewise classification rates per class (average Rec_M_ of 0.887, std 0.057). Some of the confusions between classes might be explained by similar visual appearances of the key poses (e.g., ED2 and ST4). Accumulating the classified key poses over a sequence by majority voting increases the classification performance (average Rec_M_ of 0.967, std 0.028, compare Figure 6B), but requires to analyze all frames of a sequence and is thus not well suited for real time applications. As a compromise between classification speed and performance, a sliding window based approach was evaluated. In Figure 6C, the best and worst average per class recall is displayed together with the Rec_M_ for window sizes of *n* = [1, …, 60], each with an overlap of *n* − 1. In addition, the relative number of windows which contain at least one key pose (and thus allow a classification) is shown. Table 2 summarizes the classification performance for different single frame and temporal integration schemes. Single frame performance is, in addition, reported for the evaluation of not only the key pose but the full set of frames. As can be seen, the classification performance decreases significantly but the average recall of Rec_M_ of 67.56 (std 6.06) indicates, that the learned key pose representations are still rich enough to classify a majority of the frames correctly. Note, that the relative number of correct classifications clearly exceeds the percentage of key pose frames in the dataset (per class average of 7.46%, std 2.19%, compare Figure 1C).

**Table 2:** **uulmMAD classification performance**.

			**Rec_M_**	**Inf_M_**	**Mark_M_**	**MCC_M_**
Single		All frames	67.56 ± 6.06	0.703 ± 0.062	0.762 ± 0.041	0.732 ± 0.051
		Key poses	88.65 ± 5.66	0.915 ± 0.045	0.915 ± 0.043	0.915 ± 0.044
Majority	Windowed^*^	5 [4]	89.64 ± 4.97	0.919 ± 0.041	0.920 ± 0.040	0.920 ± 0.040
		10 [9]	89.98 ± 4.69	0.922 ± 0.039	0.922 ± 0.039	0.922 ± 0.039
		20 [19]	90.47 ± 4.48	0.924 ± 0.038	0.924 ± 0.037	0.924 ± 0.037
		Full sequence	96.73 ± 2.84	0.981 ± 0.014	0.970 ± 0.033	0.975 ± 0.021

* Size [overlap]

**Figure 7:**
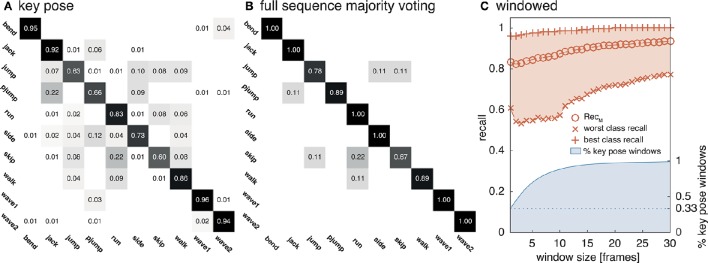
**Weizmann classification performance**. The network was evaluated on the Weizmann dataset to allow a comparison to other approaches. As in Figure 6, **(A)** shows the classifications rates for a classification of single key pose frames per class. **(B)** Displays classwise recognition results for a full sequence evaluation using majority voting. Similar visual appearances might explain the increased rate of confusions for some of the classes (e.g., *run* and *skip*). In **(C)** the average best and worst per class recall values and Recl_M_ are reported for temporal window sizes between 1 and 30 frames together with the relative number of windows which contain at least one frame classified as key pose.

The model was additionally trained using the Weizmann dataset (Gorelick et al., 2007, see Section 4.2). 1, 873 frames (per class average 187.30, std 59.51) were selected as key pose frames utilizing the combined criterion developed in Section 3.1. Except for the number of output features encoding each class (806), the same network and learning parameters were applied. As for the uulmMAD dataset, Figure 7 gives an overview over the classification performance, by showing confusion matrices for single key pose frame evaluation (Figure 7A), full sequence majority voting (Figure 7B), as well as best and worst class recall for different sized windows of temporal integration (Figure 7C). In comparison to the results reported for the uulmMAD dataset, the gap between the best and worst class recall is considerably increased. This might be explained by a different overall number of available training examples in the datasets (the per class average of training examples in the uulmMAD dataset exceeds the Weizmann dataset by a factor of 11.02), higher visual similarities between the classes (the most prominent confusions are observed for *skip, jump* and *pjump*), the lack of a sufficient number of descriptive key poses, or a combination hereof. A direct relationship of the classwise performance and the per class number of key pose frames available for training cannot be observed. Even though the least number of key pose frames was extracted for the class *bend*, the second best recall value was achieved. As for the uulmMAD dataset, performance measures are reported for different single frame and temporal integration schemes in Table 3. Again, the trained key pose representations achieve a considerable performance even when tested per frame on all frames of the action sequences (Rec_M_ = 77.15, std 6.46). Table 4 compares the reported classification results on the Weizmann dataset to state-of the art single frame based (second block) and sequence level approaches (third block). In particular, other key pose based action recognition approaches are listed (first block). The direct comparison of different classification architectures, even when evaluated on the same dataset, is often difficult, since different evaluation strategies may have been applied. Thus, whenever possible, the number of considered classes (sometimes the class *skip* is excluded) and the evaluation strategy is listed together with classification performance and speed. Evaluation strategies are either leave-one-subject-out (LOSO), leave-one-action-out (LOAO) or leave-one-out (LOO, not specifying what is left out) cross-validation.

**Table 3:** **Weizmann classification performance**.

			**Rec_M_**	**Inf_M_**	**Mark_M_**	**MCC_M_**
Single		All frames	77.15 ± 6.46	0.810 ± 0.056	0.794 ± 0.068	0.801 ± 0.061
		Key poses	82.15 ± 5.81	0.844 ± 0.061	0.827 ± 0.070	0.835 ± 0.065
Majority	Windowed^*^	5 [4]	83.50 ± 5.12	0.877 ± 0.043	0.860 ± 0.053	0.868 ± 0.047
		10 [9]	86.40 ± 5.58	0.920 ± 0.027	0.878 ± 0.067	0.899 ± 0.044
		20 [19]	90.35 ± 7.34	0.966 ± 0.023	0.898 ± 0.093	0.930 ± 0.057
		Full sequence	92.22 ± 8.33	0.980 ± 0.023	0.879 ± 0.128	0.927 ± 0.079

* Size [overlap]

**Table 4:** **Weizmann comparison to other approaches**.

**Catagory**		**# Actions**	**Evaluation**	**fps**	**Temporal range**		
					**Sub-sequence**		**Full sequence**
					**Recall**	**# Frames**	**Recall**
Key pose	Weinland and Boyer, 2008	10	LOSO	–	–	–	93.6
	Baysal et al., 2010	9	LOO	–	–	–	92.6
	Cheema et al., 2011	9	LOO	–	–	–	91.6
	Chaaraoui et al., 2013	9	LOSO	124	–	–	92.8
	Liu et al., 2013	10	LOSO	–	–	–	**100**
Single frame	Niebles and Fei-Fei, 2007	9	LOSO	–	55	1	72.8
	Fathi and Mori, 2008	10	LOO	0.25–5	**99.9**	1	**100**
	Schindler and van Gool, 2008	9	LOSO	–	93.5	1	**100**
	Hoai et al., 2011	10	LOSO	–	87.7	1	–
Full sequence	Jhuang et al., 2007	9	–	0.83	–	–	98.8
	Klaser et al., 2008	10	LOSO	–	–	–	84.3
	Grundmann et al., 2008	9	LOAO	–	–	–	94.6
	Ikizler and Duygulu, 2009	9	LOO	–	–	–	**100**
	Bregonzio et al., 2009	10	LOSO	–	–	–	96.7
	Sun and Liu, 2012	10	LOO	–	–	–	97.8
	Beaudry et al., 2016	10	LOO	51.63	–	–	**100**
	Presented approach	10	LOSO	**1, 000**	82.2	1	92.2

*Bold values indicate maximum recall/fps values per column*.

On a sequence level, the classification performance of the proposed approach is on par with almost all other key pose based methods. Only Liu et al. (2013) achieved a noteworthy higher performance (recall of 100). It is important to stress that the compared methods substantially differ in their key pose selection procedures and thus in the underlying conceptual definition of key poses. For example, Weinland and Boyer (2008) and Liu et al. (2013) select key poses that maximize the classification performance in a validation subset of the dataset, whereas (Baysal et al., 2010; Cheema et al., 2011) select and weight candidate pose descriptors dependent on their distinctiveness with respect to the other classes contained in the dataset. In Chaaraoui et al. (2013), key poses are selected independently per class using clustering in combination with a compactness metric. All the above mentioned approaches, except the last one, rely on *inter-class* distributions of pose descriptors to identify key poses, implicitly stating that representativeness is equivalent to distinctiveness (among a known set of classes). If the task at hand is to separate an a priori defined set of actions, this seems to be the superior way of defining key poses for the establishment of temporally sparse representations of actions. On the other hand such poses always describe differences based on comparisons and do not necessarily capture characteristic poses of an action.

The presented approach follows a different principle. Certain properties of image or skeleton based pose features are assumed to co-occur with characteristic body configurations and thus are used to identify key pose frames. The feature characteristic indicating a key pose and the representations/descriptors used for the recognition of a pose do not necessarily have a close relationship. In doing so, we accept the fact that the selected poses are not guaranteed to be very distinctive and some even may occur in more than one action in exactly the same way. Key poses are assumed to be the most representative poses of a particular action, not in comparison, but in general. Nevertheless, the presented results demonstrate that a *feature-driven*, pose centered key pose selection mechanism is capable of achieving the same level of performance, without loosing generality.

Most key pose based approaches in the literature try to assign single frames of an image sequence to key pose frames with a high similarity, temporally integrate the result (e.g., by using histograms or majority voting) and perform a classification of the action on a sequence level. The result of single frame action recognition based on the extracted key poses (directly linking key poses to actions) is rarely reported. Single frame based approaches (see Table 4, second block), however, try to perform action classification using information solely extracted within one frame (two frames if optical flow is part of the descriptor) and achieve impressive results. In direct comparison, the single frame performance of the presented approach (Rec_M_ of 82.15 for key pose evaluation and 77.15 for the classification of all single frames, compare Table 3) cannot compete with the other methods, which, on the contrary, utilize all frames during learning to maximize classification performance in the test training dataset. The presented approach, however, achieves a single frame performance of Rec_M_ = 77.15 when evaluated over all frames, although in case of the Weizmann dataset only a per class average of 33.84% (std 8.63%) of all frames is used for training.

In the third block of Table 4, selected approaches performing action recognition on a sequence level using a variety of different representations and classification architectures are listed. Note that in an overall comparison, (I) due to the transfer on neuromorphic hardware, the presented approach achieves the highest processing speed^6^ while consuming a minimal amount of energy, and (II) due to fact, that we aim at executing the model on a single TrueNorth chip we only use input maps with a resolution of 32 × 32 (using 4,064 of the 4,096 cores available on one chip). This is no limitation of the employed *Eedn* framework, which allows to realize models which run on systems with more than one chip (Esser et al., 2016; Sawada et al., 2016). An increased input resolution, as well as the use of more than two flow direction and contour orientation maps might help in separating classes with a high visual similarity (e.g., *skip, jump*, and *run*).

### 5.2. Comparison to full sequence learning

To address the question whether and how the proposed classification architecture might benefit from using all frames (as opposed to only key pose frames) during training, we performed exactly the same training and testing procedure twice on the uulmMAD dataset. First, only key pose frames were presented during training, while second, all frames were provided during the training phase. Likewise, testing was performed just on the preselected key pose frames, as well as the full set of frames. Table 5 compares the average recall under the different training (rows) and testing conditions (columns) for single frame evaluation and sequence level majority voting.

**Table 5:** **uulmMAD key pose versus all frame learning**.

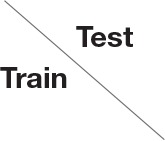	**Framewise**		**Majority voting**	
	**Key poses**	**All frames**	**Key poses**	**All frames**
**Key poses**	**88.65 ± 5.66**	67.56 ± 6.06	**96.73 ± 2.84**	93.29 ± 7.05
**All frames**	85.84 ± 7.15	72.84 ± 8.25	95.27 ± 3.93	94.70 ± 7.03

*Bold values indicate the maximum average recall for framewise and full sequence majority voting classification schemes*.

**Figure 8:**
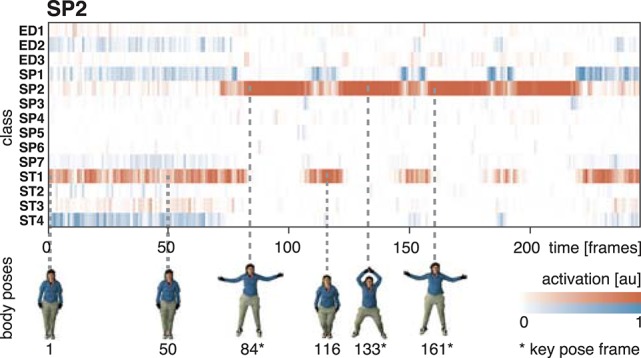
**Activation of cell populations**. The activations of the cell populations in the last convolutional layer of the DCNN assigned to the 14 classes of the uulmMAD dataset are displayed for a network trained only on key pose frames and tested on all frames of the action SP2 (jumping jack). The activation level of the cell population with the maximum activation (red) and the remaining populations (blue) is encoded by color intensity. Corresponding poses are displayed for selected frames (bottom row). Key pose frames are marked by asterisks. The activation pattern shows how the cell population assigned to the class SP2 selectively responds to frames in the temporal neighborhood of the corresponding key pose frames. At the beginning and the end of the sequence, as well as in between the occurrence of key pose frames, different cell populations achieve the maximum average activation and thus result in misclassifications. Written informed consent for the publication of exemplary images was obtained from the shown subject.

**Figure 9:**
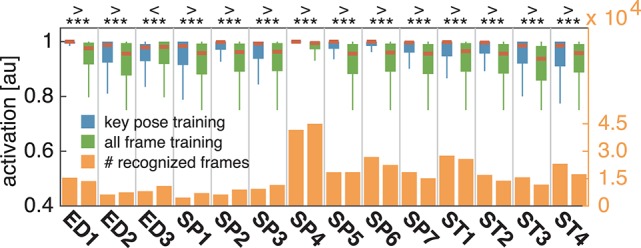
**Comparison of activation levels**. The activation levels of the cell populations which resulted in correct classifications are displayed per class for key pose based (blue) and all frame (green) training alongside with the total number of correct classifications under both conditions (yellow). In case of key pose based training, activation levels are significantly increased, reflecting a higher confidence of the classification decision. Increased confidences are useful in situations where thresholding is applied on the activation level, e.g., to reduce the number of false negatives.

In both cases, training and testing on key pose frames achieves the highest performance. However, the observed differences between the two training conditions could not shown to be significant, neither when testing on key poses nor on the full set of frames. Nevertheless, having a closer look at the activation patterns of the network reveals some insights on the effectiveness of the two variants of trained representations. Figure 8 shows the average activation pattern of the 14 cell populations in layer 15 assigned to the individual classes of a network trained on key pose frames and tested on all frames of the action SP2 (jumping jack). The displayed activation levels clearly show how the trained representations of the corresponding class selectively respond within the temporal neighborhood of the key pose frames. Frames sampled from periods without the presence of key pose frames (at the beginning and the end of the sequence, as well as in between key pose frames) result mostly in a large activation of other cell populations and thus in misclassifications. This is in line with the results shown in Table 5, which indicate that classification performance increases under both training conditions when testing is only performed on key pose frames. At this point we can conclude that, compared to a training on the full set of frames, key pose based learning of class specific representations at least performs at an equal level. Whether there is any benefit of training exclusively on key pose frames next to an increased learning speed, remains, however, an open question. Figure 9 summarizes the per class activation levels of the cell populations which resulted in a correct classification. For almost all classes (except ED3), the activation level is significantly increased when training was performed on key pose frames only. This might become a very important property in situations where it is not an option to accept any false negatives. Applying a threshold on the activation levels would allow to eliminate false negatives, while key pose based training would decrease the number of positive examples rejected by the fixed threshold. Thus, thresholding might further increase the performance for the key pose based training reported so far. Taken together, key pose based learning achieves a slightly increased classification performance with an increased selectivity of the cell populations and thus a higher confidence of the classification decisions.

### 5.3. Cross-dataset evaluation

Learning to classify input samples and the associated representations is conducted with the aim to robustly predict future outputs and, thus, generalize for new input data. Here, we evaluate such network capability by evaluating the classification of the trained network using input data across different datasets. More precisely, cross-dataset evaluation was performed to evaluate how the learned representations generalize over different datasets. The preselected key pose frames of the uulmMAD and the Weizmann dataset were used for both training and testing constellations. Performance is reported for two classes, one being *one-handed wave* (ED1 and *wave1*), which is available in both datasets. The second class was formed by combining the visually similar classes SP2/SP6 and *jack*/*wave2* during evaluation into one joint class *raising two hands*. Training was performed on the full set of classes in both cases. Thus, for *one-handed wave* a random guess classifier would achieve a recall of either 7.14 (uulmMAD) or 10.00 (Weizmann). In case of the combined class *raising two hands*, the recall chance level increases to 14.29 (uulmMAD) and 20.00 (Weizmann), respectively. Table 6 shows the result for *one-handed wave* for the two testing (row) and training (column) setups alongside with exemplary pictures of the classes from both datasets. When training was performed on the Weizmann dataset, the recall performance for examples from the uulmMAD dataset is still considerable (loss of 24.07). Training on the uulmMAD and testing on the Weizmann dataset results in an increased performance loss, but still achieves a recall of 53.03.

**Table 6:** **Cross-dataset evaluation one-handed wave**.

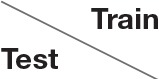	**uulmMAD**	**Weizmann**	**Loss**	
**uulmMAD**	**100**	75.93	24.07	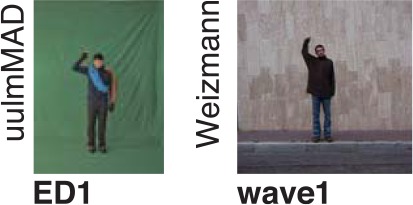
**Weizmann**	53.03	**100**	46.97	

*Bold values indicate the maximum recall values per column. Written informed consent for the publication of exemplary images was obtained from the shown subjects (uulmMAD)*.

In case of the combined class *raising two hands*, the performance loss is below 30 for both training and testing configurations. Table 7 shows the achieved performance in detail for each of the four classes in isolation and their combination. Note that when trained on the uulmMAD dataset, *jumping jack* is recognized almost without any loss of performance. Vice versa, SP2 is often confused with *wave2* when training was performed on the Weizmann dataset. This may be explained by the large visual similarities between the classes.

**Table 7:** **Cross-dataset evaluation raising two hands**.

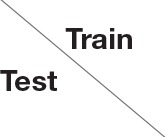		**uulmMAD**			**Weizmann**			**Loss**	
		**SP2**	**SP6**	**Comb**	***jack***	***wave2***	**Comb**	**Comb**	
**uulmMAD**	SP2	**96.97**	0.00	97.92	24.65	51.04	70.30	27.62	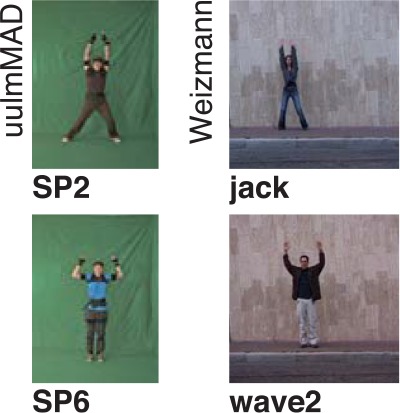
	SP6	1.14	**97.73**		0.00	64.90			
**Weizmann**	*jack*	95.96	0.00	79.80	**100**	0.00	100	20.20	
	*wave2*	31.82	31.82		0.00	**100**			

*Bold values indicate the maximum recall values per column. Written informed consent for the publication of exemplary images was obtained from the shown subjects (uulmMAD)*.

The proposed approach shows promising generalization capabilities, which might partially be explained by the class-independent, *feature-driven* selection of the key pose frames.

## 6. Conclusion and discussion

The presented work consists of two main contributions. First, a *feature-driven* key pose selection mechanism is proposed, which builds upon evidences about human action perception. The selection mechanism does not utilize any information about the inter- or intra-class distribution of the key poses (or key pose descriptors) to optimize the classification accuracy. It is demonstrated, that the classification accuracy is on par with state-of-the-art key pose based action recognition approaches, while only motion and form related feature characteristics are used to select a key pose frame. Second, we propose a biologically inspired architecture combining form and motion information to learn hierarchical representations of key pose frames. We expect such hierarchical feature representations to make the recognition more robust against clutter and partial occlusions, in comparison to holistic shape representations of the full body configurations used in previous approaches. Form and motion pattern representations are established employing a neuromorphic deep convolutional neural network. The trained network is mapped onto the *IBM Neurosynaptic System* platform, which enables a computationally and energy efficient execution.

### 6.1. Relation to other work

The presented results demonstrate, that classifying actions using a minimal amount of temporal information is in principle possible. This is in line with results from other action recognition approaches. For example, Schindler and van Gool (2008) reported that actions can be successfully recognized using snippets of three or even less frames. In their work, the length of the temporal window used for the classification of an action sequence was systematically varied. The most important result was that a reliable action recognition can be achieved by only using individual snippets, i.e. up to three consecutive frames in temporal order. The question whether there are special “key snippets” of frames, which are particularly useful for the recognition of an action and how they might be defined, however, remains open.

Inspired by evidences from perceptual studies (Thurman and Grossman, 2008; Thirkettle et al., 2009), key poses are potential candidates for representing such special events in articulated motion sequences. Unlike the majority of other approaches reported in the literature (e.g., Baysal et al., 2010; Liu et al., 2013), the proposed key pose selection mechanism identifies key pose frames without optimizing the *inter-class* distinctiveness or classification performance of the selected key poses. The *feature-driven* selection criterion proposed in this work combines form and motion information and allows the identification of key poses without any knowledge about other classes. It extends a previous proposal utilizing local temporal extrema in the motion energy as a function of time (Layher et al., 2014) by additionally taking a measure of extendedness of the silhouette shape into account. Given that these features are entirely data-driven, this has two major implications. On the one hand, the selected poses are independent of any other class and thus are more likely to generalize over different sets of actions. This property is appreciated and valuable in many applications since it does not require any prior knowledge about the distribution of classes/poses in other datasets. On the other hand, there is no guarantee, however, that a learned key pose representation is not part of more than one action and thus results in ambiguous representations. This may lead to drawbacks and deteriorations of the model performance in terms of classification rates for rather ambiguous sequences with similar pose articulations. We argue that, although the proposed key pose selection criterion might not result in the best classification performance on all action recognition datasets in isolation, it selects key pose frames which capture the nature of an action in general (independent of a specific dataset). In addition, the reported results demonstrate, that there is no substantial loss in performance when comparing the proposed *feature-driven* key pose selection mechanism to performance optimizing key pose approaches in literature. In contrast to other action recognition approaches building upon convolutional neural networks, the proposed model does not aim at establishing representations which capture the temporal relationship between successive frames. This can be accomplished by e.g., directly feeding spatiotemporal input to the network and applying 3D convolutions (e.g., Baccouche et al., 2011; Ji et al., 2013) or by applying a *multiple spatio-temporal scales neural network* (MSTNN; Jung et al., 2015). Instead, in this work, the employed DCNN exclusively aims at identifying class specific key pose frames as events in an image (and optical flow) stream.

The investigation reported in this work adds an important piece to the debate of how representations for action sequence analysis might be organized. Some previous approaches have utilized motion and form information for the classification of action categories. For example, Giese and Poggio (2003) proposed that biological motion sequences representing articulated movements of persons is subdivided into two parallel streams in primate visual cortex. In particular, the authors argue that motion patterns are represented in a hierarchy and these are paralleled by regular temporal sampling of static frames from the same input sequence. This model architecture has been extended in Layher et al. (2014) suggesting that instead of representing sequences of static frames only key poses need to be selected. As a candidate criterion, the motion energy is calculated over time and local energy minima depict reversal points of bodily articulation. Such reversals, in turn, most likely coincide with extremal articulations and thus can be utilized to select a key pose in such articulation sequences. While these models focus on cortical architecture of visual dorsal and ventral streams, other computer vision approaches also consider combinations of motion and form information for action recognition. While the proposal of Jhuang et al. (2007) builds on a hierarchy of cascaded form and motion representations, the approach of Schindler and van Gool (2008) also utilized two parallel streams of motion and form processing. Both streams generate feature vectors of equal length which are subsequently concatenated including a weighting of the relative strength of their contribution. An evaluation of the relative weights showed that a fusion with 70% motion against a 30% form feature concatenation yielded the best performance on the Weizmann dataset. On the contrary, Schindler et al. (2008) demonstrated that emotion categories can be classified using static images only which are processed by a multi-scale bank of filters with subsequent pooling operation and dimension reduction. Our findings add new insights to the investigation of utilizing form/shape and motion information in biological/articulated motion analysis for action recognition. Our findings highlight that key poses defined by events of temporal extrema in motion energy and dynamic object silhouette features reliably reflect a high information content regarding the whole action sequence. In other words, key poses can be detected by an entirely *feature-driven* approach (without utilizing any a priori model of actions in the sequence) and that the associated temporal events contain a high proportion of the information about the main components of the action sequence.

We successfully trained a DCNN of 15 convolutional layers on the key pose frames used as input, which were assigned to different action classes. The network was trained using the *energy-efficient deep neuromorphic networks* (*Eedn*) framework (Esser et al., 2016) and executed on a TrueNorth NS1e board (Merolla et al., 2014). The results show that action recognition can be performed on mobile robotic platforms under real-time constraints while consuming a minimal amount of energy. The reduced energy consumption and the high performance in classification rate (compare Table 4) makes such a model architecture a valuable candidate for applications in mobile or remote control scenarios in which autonomy in energy supply and external control are constraints of core importance. The automatic selection of key pose information for the classification mechanism is a key step to make use of the demonstrated parameters.

Although some classes contained examples with highly similar visual appearances, the network shows an impressive single frame recognition performance when tested on key frames. Even when tested on the full set of frames, recognition performance is still significantly above chance level. Using a simple temporal integration scheme, we show that the results are on par with competing key pose based action recognition approaches (Table 4). Cross-dataset evaluation of classes with the same/a similar visual appearance in both datasets shows how the learned representations generalize over the different datasets (training was performed on the full set of classes).

### 6.2. Shortcomings and possible further improvements

Currently, the optical flow estimation and the key pose selection are performed prior to the training and the classification of input sequences. To realize a complete neuromorphic implementation of the presented approach, optical flow can be estimated as well on neuromorphic hardware following the principles described in Brosch and Neumann (2016). A neuromorphic implementation of localizing the local extrema in the motion energy and the extendedness of a person's silhouette could be realized on top of the flow estimation process. In addition, dynamic vision sensors (e.g., iniLabs DVS128) are an option to directly feed a network similar to the proposed one with spike-based sensory streams. First attempts to realize an action recognition system using such sparse asynchronous data streams have already shown promising results (Tschechne et al., 2014).

The presented approach does not make use of any temporal relationship between the identified events (key poses) in an action sequence. Thus, the reversed, or scrambled presentation of images (and optical flow) of a sequence would result in an assignment to an action class, although, the visual appearance of the sequence is totally different. A modeling or learning of the temporal relationships between the key pose frames, e.g., their temporal order, would help in reducing ambiguities and thus increase sequence-wide or windowed classification rates. In case of the proposed approach, this could be achieved by employing, e.g., *long short-term memory* cells (LSTM; Hochreiter and Schmidhuber, 1997), which are candidates to realize the learning of temporal relationships without loosing the invariance against changes in speed. The simple majority voting based integration scheme was chosen, because of hardware limitations and to focus on an analysis of the importance of key poses in the context of action representation and recognition.

We also did not apply a weighted majority voting scheme using the confidences of the frame-wise predictions or apply thresholding on the predictions. Both strategies might further increase the classification performance but again would weaken the focus on the analysis of key pose base representations of action sequences.

The proposed architecture of a deep convolutional neural network (DCNN) as depicted in Figure 3 builds increasingly more complex feature representations through learning from initial simple features. It would be interesting to investigate the feature selectivities of the feature representations that have been established by the learning. Such a study would potentially shed light about the structure of the feature compositions (and their hierarchical organization) which lead to the selectivity of the key poses in relation to the action sequences to be classified. Some approaches analyzing the low-, intermediate-, and higher-level feature representations have recently been proposed in the literature (Zeiler and Fergus, 2014; Güçlü and van Gerven, 2015; Mahendran and Vedaldi, 2016). Such approaches have so far investigated CNNs for static inputs only. For that reason, some principles might also be useful for the analysis of key pose representations. In addition, the consideration of short-term spatio-temporal feature representations will help to extend the scope of the overall study of visualizing internal representations after learning. We expect necessary major efforts to carefully develop an extended set of tools which is beyond the scope of the modeling investigation presented here.

Overall, the presented results show, that the learned key pose representations allow the classification of actions using a minimal amount of temporal information. By implementing the proposed DCNN on the TrueNorth chip, we show that real-time action recognition relying on the proposed principles is possible while consuming a minimal amount of energy, as reported for the runtime environments of the *IBM Neurosynaptic System* (Esser et al., 2016).

## Author contributions

Conceived and designed the approach: GL, TB, HN; Implemented the architecture: GL, TB; Performed the simulations: GL; Analyzed the data: GL; Wrote the paper: GL, HN.

## Funding

This research has been supported by the Transregional Collaborative Research Centre SFB/TRR 62 “A Companion Technology for Cognitive Technical Systems” funded by the German Research Foundation (DFG). HN has been supported by the collaborative project “SenseEmotion” funded by the German Federal Ministry of Education and Research (BMBF).

### Conflict of interest statement

The authors declare that the research was conducted in the absence of any commercial or financial relationships that could be construed as a potential conflict of interest.

## Footnotes

^1^In the presented simulations, the MATLAB® implementation of the *Lucas-Kanade* flow estimation algorithm was used.

^2^The values of σ were chosen manually to take different temporal resolutions into account.

^3^In Figure 3, the weight matrices in the convolutional layer 5 have a dimensionality of 3 × 3 × 8, since they receive input from 256 feature maps in layer 4 which are separated into 32 groups of filters, each receiving input from 8 feature maps.

^4^Available via https://www.uni-ulm.de/imagedb.

^5^Available via http://www.wisdom.weizmann.ac.il/~vision/SpaceTimeActions.html.

^6^Image preprocessing and key pose selection is not integrated in the estimated processing time. Optical flow estimation can be performed on a second TrueNorth chip (Brosch and Neumann, 2016).
